# Depression across the menopausal stages in the United Arab Emirates: prevalence and risk factors

**DOI:** 10.3389/fgwh.2026.1702610

**Published:** 2026-02-27

**Authors:** Saumyaa Vohra, Aryan Gulati, Shivani Vohra, Anusha Sreejith

**Affiliations:** 1 MBBS Program, Gulf Medical University, Ajman, United Arab Emirates; 2 Department of Obstetrics and Gynecology, Al Qassimi Women and Children Hospital, Sharjah, United Arab Emirates; 3 Department of Community Medicine, College of Medicine, Gulf Medical University, Ajman, United Arab Emirates

**Keywords:** perimenopause, postmenopause, depression, smoking, nulliparity, hormonal contraception

## Abstract

**Background:**

Menopause amongst women in the UAE may increase the risk of depression. However, scientific data from the Middle East is limited to prove it.

**Methods:**

We conducted a cross-sectional study among 279 peri- and post-menopausal women living in the UAE (June-September 2024) using the PHQ-9 to assess depressive symptoms. Data was analyzed using SPSS version 28. Multinomial logistic regression was used to determine significant associated risk factors for depression among menopausal women.

**Results:**

10% of participants had severe depression, while 56.6% of participants had at least mild depression. Compared to postmenopausal women, perimenopausal women were more likely to experience severe depression (OR = 6.0, *p* < 0.001). Additionally, women under 50 years of age were at greater risk of depression (OR = 7.42, *p* < 0.001). Significant associations were observed with participants who had lower education (OR = 12.2, *p* < 0.001), nulliparity (OR = 3.44, *p* = 0.012), poor income (<AED 4,000) (OR = 5.32, *p* = 0.003), and hormonal contraceptive use (OR = 4.43, *p* = 0.001). Lifestyle factors like physical inactivity (OR = 4.0, *p* = 0.001), smoking (OR = 13.66, *p* < 0.001), and poor sleep quality (OR = 7.96, *p* < 0.001) were also significantly associated with severe depression.

**Conclusion:**

Depression is highly prevalent among women going through menopausal transition in the UAE, with perimenopausal women and modifiable risk factors (smoking, physical inactivity, poor sleep quality) demonstrating significant associations. These results reinforce the need for early mental health screening and lifestyle counseling for women in the UAE, especially those going through the menopausal transition.

## 1 Introduction

Menopause is the phase in a female's life when ovarian function comes to an end, leading to permanent cessation of menstruation. The World Health Organization (WHO) defines natural menopause as occurring after 12 consecutive months of amenorrhea, typically around 51.4 years of age (1). This stage is physiologically universal and signifies a critical transition in women's health.

This period, also known as post menopause, results primarily from the near-complete depletion of ovarian follicles, causing decreased estrogen levels and increased follicle-stimulating hormone (FSH) levels. On the other hand, the menopausal transition, or perimenopause, occurs after a woman's reproductive years and is marked by irregular menstruation, hormonal changes due to decreased sensitivity to gonadal steroid feedback, and symptoms such as hot flashes. During this transitional stage, significant hormonal fluctuations can make women more vulnerable to major depression (2).

There is substantial evidence linking menopause and depression. The International Classification of Diseases (ICD-11) defines a depressive episode as a period of markedly diminished interest in activities or a depressed mood lasting almost every day for at least two weeks. The symptoms of depression can significantly impair daily functioning at home, work, or in social settings (3).

Studies show that during perimenopause, 45% to 68% of women experience depressive symptoms, significantly impacting quality of life, increasing healthcare costs, and causing considerable suffering. Identifying factors associated with an elevated risk of depression can help women and their physicians understand the likely course of depression during menopause and improve therapeutic approaches (4).

Reduced estrogen has been particularly linked to psychosomatic symptoms in menopausal women including depression (5). Serotonin, a neurotransmitter associated with happiness and well-being, is influenced by menstrual cycle-regulating hormones. As these hormone levels drop during menopause, so does the serotonin level, potentially leading to increased risk of depression (2). However, findings remain inconsistent. Ghorbani et al. reported no correlation with estrogen but a significant association between vitamin D levels and depression in menopausal women (5).

Women who experience significant depressive episodes during menopause are often more likely to have had previous mood episodes. Menopause at an older age appears protective. Research suggests that menopause attained at 40 years or older is associated with a 50% lower risk of later-life depression compared to those who attained menopause before 40 years of age. Additionally, each two-year increase in menopausal age confers an additional 5% reduction in severe depression risk. This suggests that longer lifetime exposure to estrogen is linked to a decreased risk of depression in later life (4).

Ahlawat et al. found that 41.6% of postmenopausal women experienced depression, with higher prevalence among younger women, those of lower socioeconomic status, and women with low educational level. Depression was also more common among divorced or widowed women (1). Similarly, Nobahar et al. reported that postmenopausal women were more likely to screen positive for depression compared to premenopausal women. Women with menopausal symptoms had higher rates of depression in the year before and after menopause (6).

In 1990, approximately 467 million women worldwide were postmenopausal. This number is expected to double by 2030, reaching 1.2 billion, with 47 million women entering menopause annually. The dramatic increase in the number of menopausal women highlights the urgent public health importance of studying menopause and its impact on women's mental health (7).

Various studies have examined factors influencing depressive symptoms in postmenopausal women, focusing on symptom management, longitudinal changes, hormones, and influencing variables in older menopausal women (8–10). However, comparative research of depression across perimenopausal and postmenopausal stages is limited and data from the Middle East is virtually absent. This is a critical gap, especially given the sociocultural, economic, and lifestyle factors unique to this population that may shape mental health outcomes.

Thus, the objective of the current research was to estimate the prevalence of depression and to assess associated risk factors including sociodemographic, reproductive and lifestyle factors among menopausal women in the UAE. Addressing this gap in evidence is important to guide culturally sensitivity interventions and provide relevant information to women's mental health policies in rapidly developing regions.

## 2 Materials and methods

### 2.1 Study design and settings

We conducted a cross-sectional study among peri and postmenopausal women residing in UAE between June 2024 and September 2024.

### 2.2 Participants

Eligibility criteria included women above 40 years old being in perimenopause or post menopause period and residing in the UAE. Exclusion criteria was women who had undergone hysterectomy and/or bilateral oophorectomy, women unable to respond to the questionnaire due to cognitive impairment and those who had already been diagnosed with depression.

### 2.3 Data collection and ethics

Data were collected using a standardized, validated self-administered online questionnaire circulated as google forms. A pilot study was conducted among five participants to assess clarity and reliability.

A non-probability convenience sampling method was used to recruit women aged 40 years and above across the United Arab Emirates. Majority of the recruitment was conducted through direct in- person approach by investigators at hospitals and outpatient clinics. Women who appeared to be withing the menopausal age group were approached, and the study purpose was explained to them in brief. Those who agreed to participate were handed over a QR code that linked directly to the online questionniare, which they completed on their own phones/devices. Additional participation was promoted through social media platforms like whatsapp, facebook and reddit, where the questionnaire's link was shared to a wider audience. Eligibility criteria included women aged ≥40 who were postmenopause or undergoing perimenopausal symptoms. Women who had attained surgical menopause by undergoing hysterectomy and/or bilateral oophorectomy were excluded.

A total of 334 women above 40 years were approached during the data collection period. Of these, 44 were excluded as they had not yet reached menopause, 9 were excluded due to surgical menopause, and 2 were excluded due to missing data.

This sampling method allowed for wide geographic reach and participation but had limited generalizability as only women who had internet access and were more health literate were able to participate.

Ethical approval for the research was attained from the IRB of Gulf Medical University in Ajman, United Arab Emirates. Data from the participants were taken only after getting consent from them.

### 2.4 Variables

The questionnaire included questions on the prevalence and scaling of depression among menopausal women using the Patient Health Questionniare-9 (PHQ-9) (11). This questionnaire is a validated screening tool for depression that measures symptom frequency over the previous 2 weeks across 9 DSM-IV criteria. The internal consistency of the PHQ-9 in our sample was Cronbach's Score α = 0.87, indicating high reliability. Scores were classified as minimal (≤4), mild to moderate (5–14), and moderately severe to severe (15–27). Participants in this study with minimal scores (≤4) were considered to have normal mood with no functional impairment, while those with mild to moderate scores (5–14) had subclinical or probable depression. Lastly, women with moderately severe to severe scores (15–27) had a higher likelihood of major depressive disorder requiring clinical intervention.

Postmenopause was defined as ≥12 months of amenorrhea after age 40; perimenopause was defined as irregular cycles with menopausal symptoms after age 40.

In addition to this, information related to potential factors associated with depression were also collected, including sociodemographic factors, Body Mass Index (BMI), marital status, employment status, monthly income, status of hormonal therapy use, history of chronic diseases, sleep pattern, physical activity, history of mental health disorder, alcohol and smoking status.

To minimize bias, we used standardized questionnaires, ensured anonymity, and applied uniform data collection procedures.

### 2.5 Study size

The sample size was calculated based on a prevalence rate of 29%, derived from a study (12) using the formula S = z^2^pq/L^2^ for a cross-sectional study.

*p* = 0.29, q = 0.71, z = 1.96, L = 0.05.

The total calculated sample size was 348. We approached 334 participants over the age of 40 during the data collection period. Out of these, 44 were excluded as they had not yet reached menopause, 9 were excluded as they had attained surgical menopause, and 2 responses were removed due to missing data. Eventually, 279 valid responses were obtained, giving a response rate of 83.5%. The sample size achieved (*n* = 279) was slightly lower than the calculated requirement (*n* = 348, which may have decreased the statistical power to identify weaker associations between variables in the study. Although this reduction in sample size could modestly affect external validity, the results remained consistent with findings from previous studies within the region, which makes the results reliable. Nonetheless, findings should still be interpreted with caution, considering that the smaller sample size could limit generalizability of conclusions to the wider population.

### 2.6 Statistical methods

Data were analyzed using SPSS version 28. The chi-square test was employed to identify associations between depression in menopause and other factors. Multinomial logistic regression analyses were conducted to determine significant factors associated with depression among menopausal women. Confounding factors were controlled by including them in the regression models. The significance level was set at *P* < 0.05.

## 3 Results

**Figure 1:**
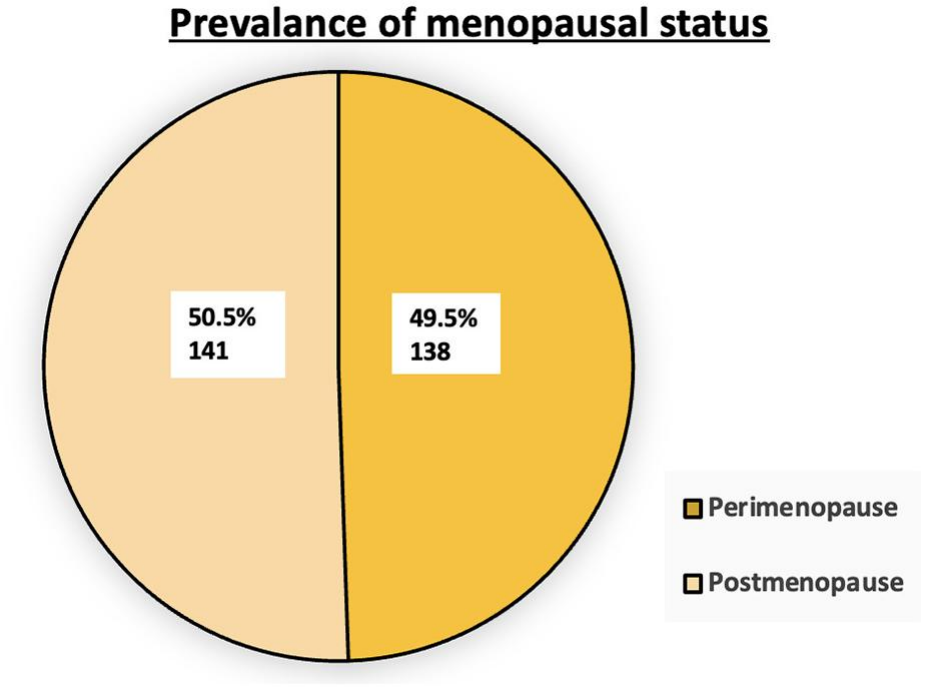
Distribution of menopausal status among participants (*N* = 279).

Figure 1 shows that 138 (49.5%) participants were perimenopausal and 141 (50.5%) were postmenopausal. The sample was approximately equally divided between the two groups.

**Figure 2:**
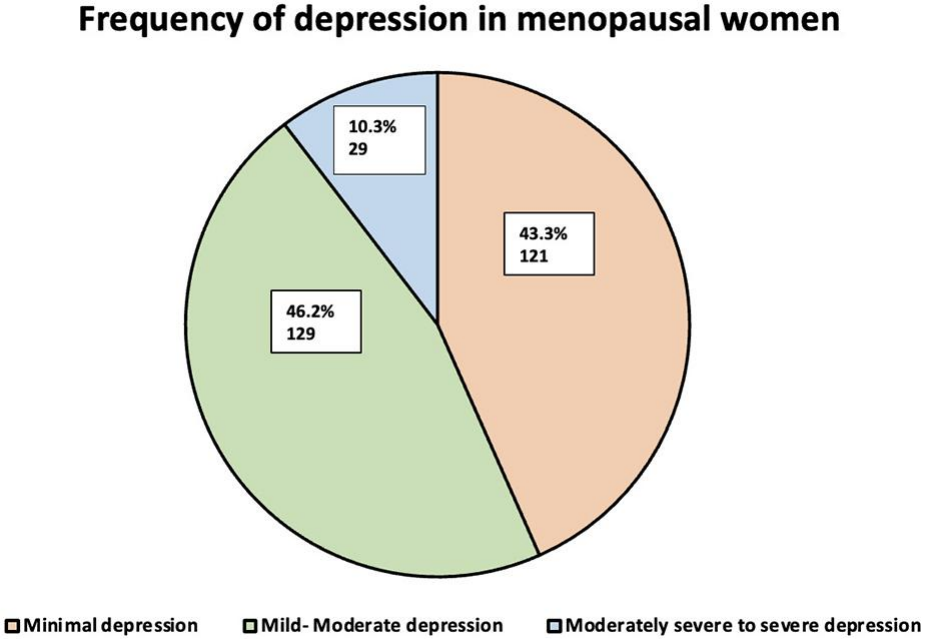
Distribution of depression severity among menopausal women, evaluated using the PHQ-9 scale (*N* = 279).

Figure 2 shows that minimal depression was seen in 121 women (43.3%), mild to moderate depression was seen in 129 (46.2%) women, and moderately severe to severe depression was seen in 29 women (10.3%).

Table 1 shows that women aged ≤50 years were significantly more likely to experience severe depression (19.5%) compared to those >50 years (19.5% vs. 4.2%, *p* < 0.001). Lower educational level (high school/undergraduate) was strongly associated with higher prevalence of moderately severe to severe depression (18.3%) compared to women with postgraduate education (3.4%, *p* < 0.001). Nulliparity was also a significant factor that was associated with severe depression (16.4% vs. 8.9% in parous women, *p* = 0.009). Lower monthly income (<4,000 AED) was linked to higher depression severity (*p* = 0.024).

**Table 1:** Sociodemographic factors and their association with depression severity among women in the UAE (*N* = 279).

Socio-demographic variables		Depression			*P*-value
		Minimal depression	Mild-moderate depression	Moderately severe- severe depression	
Age	≤50 years	36 (31.9)	55 (48.7)	22 (19.5)	<0.001
	>50 years	85 (51.2)	74 (44.6)	7 (4.2)	
Education	High school/Undergrad	34 (26.0)	73 (55.7)	24 (18.3)	<0.001
	Postgrad and above	87 (58.8)	56 (37.8)	5 (3.4)	
Relationship status^a^	Single	8 (42.1)	8 (42.1)	3 (15.8)	–
	Married/Living in	106 (45.9)	102 (44.2)	23 (10)	
	Divorced/Widowed	7 (24.1)	19 (65.5)	3 (10.3)	
Employment status	Currently Employed	79 (45.7)	77 (44.5)	17 (9.8)	0.610
	Currently unemployed	42 (39.6)	52 (49.1)	12 (11.3)	
Monthly income	Less than 4,000 AED	18 (32.7)	27 (49.1)	10 (18.2)	0.024
	4,000–10,000 AED	36 (43.9)	34 (41.5)	12 (14.6)	
	More than 10,000 AED	67 (47.2)	68 (47.9)	7 (4.9)	
Living status^a^	Living alone	12 (28.6)	23 (54.8)	7 (16.7)	–
	Living with family	109 (46)	106 (44.7)	22 (9.3)	
Number of children	No	14 (25.5)	32 (58.2)	9 (16.4)	0.009
	Yes	107 (47.8)	97 (43.3)	20 (8.9)	

^a^ Chi square was not valid as expected frequency was less than 5.

Table 2 shows that depression severity differ significantly by menopausal status. Among perimenopausal women, 68 out of 138 (49.3%) reported mild/moderate depression and 23 (16.7) reported severe depression. On the other hand, among postmenopausal women, 61 out of 141 (43.3%) had mild/moderate symptoms and 6 (4.3%) reported severe depression symptoms (*p* < 0.001).

**Table 2:** Association between menopausal status and depression severity (*N* = 279).

Menopausal status		Depression			*P*-value
		Minimal depression	Mild-moderate depression	Moderately severe- severe depression	
Menopausal status	Perimenopause	47 (34.1)	68 (49.3)	23 (16.7)	<0.001
	Post menopause	74 (52.4)	61 (43.3)	6 (4.3)	

Table 3 shows that lower physical activity (≤2 times/week) was significantly associated with higher prevalence of severe depression (12.5%) compared to those participants who engaged in more frequent physical activity (>2 times/week; 5.2%; *p* = 0.002). Smoking status (current or former) was significantly associated with severe depression (21.6% vs. 6.3% in never smokers, *p* < 0.001).

**Table 3:** Association between contraceptive history, lifestyle factors and depression severity (*N* = 279).

Lifestyle factors		Depression			*P*-value
		Minimal depression	Mid-mod depression	Moderately severe- severe depression	Total
Frequency of physical activity	Equal to or less than 2 times a week	38 (31.7)	67 (55.8)	15 (12.5)	0.002
	More than 2 times a week	62 (53.4)	48 (41.4)	6 (5.2)	
Smoking status	Never smoker	111 (51.4)	81 (39.5)	13 (6.3)	<0.001
	Current and former smoker	10 (13.5)	48 (64.9)	16 (21.6)	
Alcohol drinking status	No	73 (47.1)	68 (43.9)	14 (9.0)	0.339
	Yes	48 (38.7)	61 (49.2)	15 (12.1)	
Contraceptive history	No	76 (55.5)	53 (38.7)	8 (5.8)	<0.001
	Yes	45 (31.7)	76 (53.5)	21 (14.8)	
Sleep duration	≤6 h	52 (38.2)	66 (48.5)	18 (13.2)	0.137
	>6 h	69 (48.3)	63 (44.1)	11 (7.7)	
How long does it take to fall asleep	Less than 15 min	48 (57.8)	30 (36.1)	5 (6.0)	0.003
	15–30 min	47 (43.1)	53 (48.6)	9 (8.3)	
	More than 30 min	26 (29.9)	46 (52.9)	15 (17.2)	
How often do you wake up at night after falling asleep?	≤1 time per night	91 (56.2)	63 (38.9)	8 (4.9)	<0.001
	≥2 times per night	30 (25.6)	66 (56.4)	21 (17.9)	

Women with a history of contraceptive use were more likely to report severe depression (21 out of 142,14.8%) compared to those without contraceptive use (8 out of 137, 5.8%). The association between contraceptive history and depression severity was statistically significant (*p* < 0.001).

Longer sleep latency (>30 min) and frequent night awakenings (≥2 times per night) were significantly associated with higher rates of severe depression (17.2% and 17.9%, respectively). Both associations were statistically significant (*p* = 0.003 and *p* < 0.001, respectively).

Table 4 shows the unadjusted multinomial logistic regression which demonstrates that younger age (≤50 years), lower education, nulliparity, perimenopausal status, contraceptive use, physical inactivity, smoking, longer sleep latency, and frequent night awakenings were significantly associated with both mild-moderate and severe depression.

**Table 4:** Unadjusted multinomial logistic regression analysis of factors associated with depression severity (*N* = 279).

Variable		Mild-moderate depression			Moderately severe to severe depression		
		OR	95% CI	*P*-value	OR	95% CI	*P*-value
Age	≤50 years	1.76	1.04–2.96	0.035	7.42	2.91–18.9	<0.001
Education	high school/undergrad	3.3	1.97–5.65	<0.001	12.2	4.33–34.8	<0.001
Income	<4,000 AED	1.48	0.75–2.93	0.264	5.32	1.78–15.93	<0.003
	4,000–10,000 AED	0.93	0.52–1.66	0.807	3.2	1.56–8.82	0.025
Children	No children	2.52	1.27–5.0	0.008	3.44	1.31–9.02	0.012
Menopausal status	`Perimenopause	1.76	1.06–2.90	0.028	6.04	2.29–15.92	<0.001
Contraceptive history	Yes	2.42	1.46–4.03	<0.001	4.43	1.81–10.84	0.001
Physical activity	Equal to or less than 2 times a week	2.28	13.16–3.94	<0.003	4.08	1.46–11.42	0.001
Smoking status	Current and former smoker	6.6	3.14–13.8	<0.001	13.66	5.14–36.3	<0.001
How long does it take to fall asleep	15–30 min	2.83	1.46–5.49	0.002	5.54	1.81–16.96	0.003
	>30 min	1.80	0.99–3.29	0.055	1.83	0.57–5.89	0.306
How often do you wake up at night after falling asleep?	≥2 times per night	3.18	1.86–5.44	<0.001	7.96	3.2–19.83	<0.001

Table 5 shows that after adjustment for potential confounders, perimenopausal women had nearly six-fold higher odds of moderately severe to severe depression compared to postmenopausal women (OR 6.04, 95% CI 2.29–15.92). Women aged ≤50 years had 7.42-fold higher odds (95% CI 2.91–18.9) of severe depression. Lower education, low income, nulliparity, hormonal contraceptive use, physical inactivity, smoking, and poor sleep quality all remained independently associated with increased odds of depression. Among these, smoking (OR 13.66, 95% CI 5.14–36.3) and frequent night awakenings (OR 7.96, 95% CI 3.2–19.83) showed the strongest associations.

**Table 5:** Adjusted multinomial logistic regression of factors associated with depression severity (*N* = 279).

Variable		Mild-moderate depression			Moderately severe to severe depression		
		OR	95% CI	*P*-value	OR	95% CI	*P*-value
Age	≤50 years	1.76	1.04–2.96	0.035	7.42	2.91–18.9	<0.001
Education	high school/undergrad	3.3	1.97–5.65	<0.001	12.2	4.33–34.8	<0.001
Income	<4,000 AED	1.48	0.75–2.93	0.264	5.32	1.78–15.93	<0.003
	4,000–10,000 AED	0.93	0.52–1.66	0.807	3.2	1.56–8.82	0.025
Children	No children	2.52	1.27–5.0	0.008	3.44	1.31–9.02	0.012
Menopausal status	Perimenopause	1.76	1.06–2.90	0.028	6.04	2.29–15.92	<0.001
Contraceptive history	Yes	2.42	1.46–4.03	<0.001	4.43	1.81–10.84	0.001
Physical activity	Equal to or less than 2 times a week	2.28	13.16–3.94	<0.003	4.08	1.46–11.42	0.001
Smoking status	Current and former smoker	6.6	3.14–13.8	<0.001	13.66	5.14–36.3	<0.001
How long does it take to fall asleep	15–30 min	2.83	1.46–5.49	0.002	5.54	1.81–16.96	0.003
	>30 min	1.80	0.99–3.29	0.055	1.83	0.57–5.89	0.306
How often do you wake up at night after falling asleep?	≥2 times per night	3.18	1.86–5.44	<0.001	7.96	3.2–19.83	<0.001

## 4 Discussion

Our study found that 56.6% menopausal women had mild depressive symptoms, while 10% had severe depression using the PHQ-9 scale. Recent meta-analyses indicate that the global prevalence of depression among postmenopausal women is approximately 28%, while in developing countries it is higher, estimated at 37.3% (13). In the UAE, this burden appears even greater. For example, Hamid et al. reported that around 23% of postmenopausal women in Al-Ain, UAE, experienced mood-related symptoms (14). The higher prevalence in our study may reflect both a growing awareness regarding mental health and the influence of rapid lifestyle changes affecting women in the UAE. These figures are also notably higher than those reported in neighboring Gulf countries, such as Qatar, where prevalence rates among menopausal women range from 17.1% to 23.3%. This suggests a significant and perhaps under-recognized mental health burden among menopausal women in this region (14, 15).

Our results also demonstrated that perimenopausal women were significantly more likely to report depression compared to postmenopausal women. In specific, odds of mild to moderate depression were almost doubled, and odds of severe depression were six-fold higher. These findings are supported by previous studies, such as Badawy et al., which showed a 40% increased risk of depression in perimenopausal women relative to premenopausal women (16). Hormonal instability during the transitional phase, especially fluctuating estrogen levels, likely contributes to this risk by altering mood-regulating neurotransmitters like serotonin and dopamine (17–19).

Younger age also emerged as a strong predictor. Women aged 50 years or less had markedly higher odds of severe depression than those older than 50. This finding matches the results of a study conducted by Wu et al., who reported a 5% reduction in depressive symptoms for every 2-year delay in menopause (20). Likewise, Shao et al. also demonstrated an inverse relationship between age at which physiological menopause was attained and risk of depression (21). These findings suggest that longer lifetime estrogen exposure, which is considered neuroprotective, may lower risk of developing depression.

Socioeconomic factors were strongly associated with depression. Women with a lower monthly income were over five times more likely to have severe depression, and those with only high school or undergraduate education had a twelve-fold higher risk compared with women with postgraduate education. These associations reflect broader global findings. Rahman et al. found that lower income and education status consistently predicted menopausal mood disorders across various populations (22). Limited access to healthcare, high caregiving burdens, and financial stress are likely mediators.

Reproductive history was also significant. Nulliparous women had a threefold increased risk of depression. Similar results were exhibited by Peterson et al., which showed higher depressive symptoms among perimenopausal women with no children, particularly those who were infertile (23). This is likely due to reduced maternal role identity and reduced social support systems associated with parenting. Cultural expectations about motherhood in Arab societies may cause additional worsening of the psychological impact of childlessness during mid-life.

Current or previous use of hormonal contraception was also linked to higher depression severity. Women with a history of contraceptive use were at a fourfold higher likelihood of severe depression. This is consistent with a study by Rashid et al., which noted that hormonal contraceptives can induce depressive symptoms in hormonally sensitive women, despite many years after use (24). A review by Lewis et al. explains how certain contraceptives are associated with negative biases in emotion recognition, blunted reward responses, and dysregulated stress reactivity, especially in women with pre-existing mood vulnerability (like in menopause), which could be the reason for this association (25). Although causality is not yet established, physicians should be aware of this association when counseling women who require or are on hormonal contraception.

Lifestyle factors were also important. Physically inactive lifestyle (≤2 exercise sessions per week) was associated with a fourfold increase in severe depression. This is consistent with results from two meta-analyses that showed that moderate physical activity reduces depressive symptoms in menopausal women (26, 27). Regular physical activity has been consistently shown to increase brain-derived neurotrophic factor (BDNF) which is a protein vital for neuroplasticity. A meta-analysis from 14 human studies reported that both acute and program regular exercise significantly increased BDNF levels, signifying how exercise improves neurobiological resistance against depression (28). As a result, reduced BDNF gives an adequate explanation as to why physical inactivity increases the risk of depression in menopausal women.

Smoking emerged as one of the strongest predictors. Current or former smokers had a nearly fourteenfold greater risk of severe depression. This aligns with large cohort study and meta-analytic data reporting a dose-dependent relationship between smoking and depressive symptoms (29, 30). Nicotine related systemic inflammation and hormone dysregulation may lead to this association (31, 32).

Sleep quality was also an important independent factor. Women with delayed sleep onset (>15 min) or had two or more nighttime awakenings had five to eight times higher odds of severe depression. Similar results were reported by Meng et al., who found that poor sleep among menopausal women was an independent predictor of depression, regardless of hormone levels (33). Freedman also notes that fragmented sleep is both a symptom and a driver of depressive episodes during the menopause transition (34). In addition, a prospective cohort of 1,579 women aged 44–56 found that those experiencing sleep–wake rhythm changes over 1 year had a 51% higher risk of developing incident depressive symptoms over a 7-year follow-up, despite adjusting for other factors. This indicates that disrupted sleep may act as a causal risk factor for depression in menopausal women (35).

The sociocultural setting in the UAE may influence the prevalence of depression reported in this study. Cultural stigma, gender role expectations, and fear of social judgement are identified as major barriers to seeking mental health support among female residents in the UAE, especially women of Emirati background. This results in delayed diagnosis and underreporting of symptoms (36). Furthermore, responsibilities of family care and socioreligious expectations may further add to the emotional burden of experiencing menopause.

Taken together, these findings reinforce that depression in menopausal women is a complex interplay of hormonal, psychological, social, and quality-of-life related factors (37). Addressing this burden requires integrated primary care strategies. Routine PHQ-9 screening during gynecologic visits, especially for perimenopausal and socioeconomically disadvantaged women, could help with early detection. Physicians must also evaluate contraceptive history, encourage physical activity, offer smoking cessation support, and provide resources for improving sleep hygiene. Additionally, the need for national screening and counseling programs should be emphasized, especially through family medicine and gynecology clinics. This would encourage early detection and culturally appropriate mental health care.

This study has several limitations. Firstly, it follows a cross sectional design, hence it cannot prove a causal link between depression and associated factors. Secondly, recall bias may be introduced by self-reported data. Thirdly, majority of the study sample was taken from urban health clinics which may limit generalizability to rural or non-health-seeking populations. Additionally, selection bias may have also occurred, as only literate women were more likely to participate, leading to underrepresentation of women with lower education. Data collection was mostly conducted through online self-administered questionnaires, so women with limited access to the internet may have also been underrepresented. The accuracy and completeness of some analyses may have been hampered by missing data in some questionnaire replies. Lastly, the final sample size (*N* = 279) was smaller than the calculated requirement (*N* = 348). This could have decreased the statistical power and increased the margin of error.

In summary, depression in menopausal women in the UAE is highly prevalent and closely linked to multiple modifiable risk factors. The greatest burden of depression falls on women who are perimenopausal, younger, socioeconomically disadvantaged, nulliparous, physically inactive, smokers, poor sleepers, or with a history of contraceptive use. As part of comprehensive middle age care for women, these findings advocate for culturally appropriate, customized mental health screening programs and treatments.

## 5 Conclusion

According to this study, menopausal women in the UAE had a significant prevalence of depressive symptoms (56.6%), with 10% experiencing severe depression. Women younger than 50, perimenopausal, nulliparous, or with lower incomes or educational levels were at the greatest risk. Severe depression was also more common in women with a history of hormonal contraceptive use.

Lifestyle habits played a key role. Women who were physically inactive, current or former smokers, and those with poor sleep quality (delayed sleep onset or frequent nighttime awakenings) were much more likely to be depressed. These findings signify that many of the risk factors are modifiable. Therefore, it is important to prioritize early mental health screening and lifestyle counseling during routine gynecologic and primary care visits for middle aged women undergoing menopause in the UAE. Physicians must also give special attention to socioeconomically disadvantaged**,** perimenopausal**,** and nulliparous women and provide them with lifestyle counseling to promote physical activity**,** smoking cessation**,** and sleep hygiene. Policymakers must also initiate menopausal mental health screening into existing women health initiatives to ensure early detection and targeted support for women within the UAE.

## Data Availability

The raw data supporting the conclusions of this article will be made available by the authors, without undue reservation.
